# (1*H*-1,2,4-Triazol-1-yl)methyl 2-(2,4-dichloro­phen­oxy)acetate

**DOI:** 10.1107/S1600536810012456

**Published:** 2010-04-21

**Authors:** Yun-Man Xie, Yu-Min Li

**Affiliations:** a Henan Chemical Industry Research Institute Co. Ltd, Zhengzhou 450052, People’s Republic of China

## Abstract

In the title compound, C_11_H_9_Cl_2_N_3_O_3_, the triazole and benzene rings are roughly parallel to one another [dihedral angle = 4.99 (2)°] because the C—O—C—C—O chain that links the two rings is folded [O—C—C—O = 8.60 (2)°] rather than fully extended. In the crystal, weak inter­molecular C—H⋯N and C—H⋯O inter­actions are present, and π–π inter­actions are indicated by the short distances [3.749 (3) Å] between the centroids of the triazole and benzene rings.

## Related literature

For details of the biological activities of triazole-containing compounds, see: Xu *et al.* (2002 ▶). For bond-length data, see: Allen *et al.* (1987 ▶).
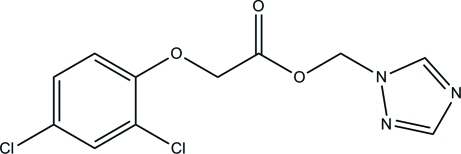


## Experimental

### 

#### Crystal data

C_11_H_9_Cl_2_N_3_O_3_
*M* *_r_* = 302.11
Monoclinic, 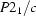

*a* = 10.814 (2) Å
*b* = 6.4514 (13) Å
*c* = 18.698 (4) Å
β = 101.05 (3)°
*V* = 1280.2 (4) Å^3^
*Z* = 4
Mo *K*α radiation
μ = 0.51 mm^−1^
*T* = 293 K
0.74 × 0.22 × 0.05 mm

#### Data collection

Rigaku R-AXIS RAPID IP area-detector diffractometer
Absorption correction: multi-scan (*ABSCOR*; Higashi, 1995 ▶) *T*_min_ = 0.702, *T*_max_ = 0.976
2253 measured reflections
2253 independent reflections
1816 reflections with *I* > 2σ(*I*)
*R*_int_ = 0.000

#### Refinement

*R*[*F* ^2^ > 2σ(*F* ^2^)] = 0.036
*wR*(*F* ^2^) = 0.096
*S* = 1.02
2253 reflections
172 parameters
H-atom parameters constrained
Δρ_max_ = 0.24 e Å^−3^
Δρ_min_ = −0.34 e Å^−3^

### 

Data collection: *RAPID-AUTO* (Rigaku, 2004 ▶); cell refinement: *RAPID-AUTO*; data reduction: *RAPID-AUTO*; program(s) used to solve structure: *SHELXTL* (Sheldrick, 2008 ▶); program(s) used to refine structure: *SHELXTL*; molecular graphics: *SHELXTL*; software used to prepare material for publication: *SHELXTL*.

## Supplementary Material

Crystal structure: contains datablocks I, global. DOI: 10.1107/S1600536810012456/hg2669sup1.cif

Structure factors: contains datablocks I. DOI: 10.1107/S1600536810012456/hg2669Isup2.hkl

Additional supplementary materials:  crystallographic information; 3D view; checkCIF report

## Figures and Tables

**Table 1:** Hydrogen-bond geometry (Å, °) *Cg*1 and *Cg*2 are the centroids of the triazole ring (C10/C11/N1–N3) and the benzene ring (C1–C6), respectively.

*D*—H⋯*A*	*D*—H	H⋯*A*	*D*⋯*A*	*D*—H⋯*A*
C6—H6*A*⋯N3^i^	0.93	2.45	3.353 (3)	164
C7—H7*B*⋯O1^ii^	0.97	2.58	3.390 (2)	142
C9—H9*A*⋯O2^iii^	0.97	2.52	3.381 (3)	148
C11—H11*A*⋯O2^iii^	0.93	2.54	3.293 (2)	139
*Cg*1⋯*Cg*2^i^			3.665 (2)	

Symmetry codes: (i) 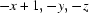; (ii) 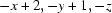; (iii) 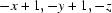.

**Fig. 1.**
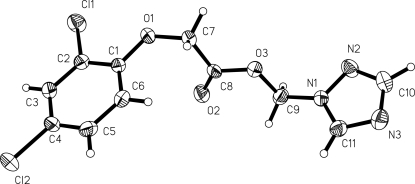
The molecular structure of (I), with atom labels and 40% probability displacement ellipsoids for non-H atoms.

## supplementary crystallographic information

### Comment

Compounds containing the triazole ring system are well known as efficient fungicides in agriculture, where they act by inhibiting the biosynthesis of ergosterol (Xu *et al.*, 2002). In order to search for new triazole compounds with higher bioactivity, the title compound was synthesized and its structure is reported here.

In title compound, all bond lengths in the molecular are normal (Allen *et al.*, 1987). Triazole ring (C10/C11/N1—N3) and benzene ring (C1—C6) are essentially parallel to one another (dihedral angle of 4.99 (2)°) because the C—O—C—C—O chain that links the two rings is folded [O—C—C—O torsion angle = 8.60 (2)°] rather than fully extended. π—π interactions are indicated by the short distance (Cg1···Cg1 distance of 3.749 (3) Å, symmetry code: 1-x,-y,-z) between the centroids of the triazole rings (C10/C11/N1—N3) (Cg1) and benzene rings C1—C6 (Cg2) (Table 1). There are weaker C—H···N, C—H···O intermolecular interactions, which stabilized the structure (Table 1).

### Experimental

To a 100 ml flask were added 10 mmol of (1*H*-1,2,4-triazol-1-yl)methanol and 11 mmol of triethylamine in 20 ml of dryed acetone, to which 10 mmol of 2-(2,4-dichlorophenoxy)acetyl chloride in 10 ml of acetone was then dropwise added with stirring on ice-cold water bath within 0.5 h. The reaction took place immediately, and a lot of white solid appeared. The mixture was heated and refluxed for 2 h, and then cooled to room temperature. After filtering and distilling in reduced pressure, a crude product was obtained and purified by flash column chromatography (silicagel, using ethyl ethanoate: cyclohexane = 1:3 as eluent) to afford the title compound. Single crystals suitable for X-ray measurements were obtained by recrystallization from ethyl acetate at room temperature.

### Refinement

H atoms were positioned geometrically and refined using a riding model, with C—H = 0.93 or 0.97 Å and with *U*_iso_(H) = 1.2 times *U*_eq_(C).

### Crystal data

**Table:**

C_11_H_9_Cl_2_N_3_O_3_	*F*(000) = 616
*M**_r_* = 302.11	*D*_x_ = 1.567 Mg m^−^^3^
Monoclinic, *P*2_1_/*c*	Mo *K*α radiation, λ = 0.71073 Å
Hall symbol: -P 2ybc	Cell parameters from 11868 reflections
*a* = 10.814 (2) Å	θ = 1.9–27.4°
*b* = 6.4514 (13) Å	µ = 0.51 mm^−^^1^
*c* = 18.698 (4) Å	*T* = 293 K
β = 101.05 (3)°	Thin platelet, colorless
*V* = 1280.2 (4) Å^3^	0.74 × 0.22 × 0.05 mm
*Z* = 4	

### Data collection

**Table:**

Rigaku R-AXIS RAPID IP area-detector diffractometer	2253 independent reflections
Radiation source: Rotating Anode	1816 reflections with *I* > 2σ(*I*)
graphite	*R*_int_ = 0.0000
ω Oscillation scans	θ_max_ = 25.0°, θ_min_ = 1.9°
Absorption correction: multi-scan (*ABSCOR*; Higashi, 1995)	*h* = 0→12
*T*_min_ = 0.702, *T*_max_ = 0.976	*k* = 0→7
2253 measured reflections	*l* = −22→21

### Refinement

**Table:**

Refinement on *F*^2^	Primary atom site location: structure-invariant direct methods
Least-squares matrix: full	Secondary atom site location: difference Fourier map
*R*[*F*^2^ > 2σ(*F*^2^)] = 0.036	Hydrogen site location: inferred from neighbouring sites
*wR*(*F*^2^) = 0.096	H-atom parameters constrained
*S* = 1.02	*w* = 1/[σ^2^(*F*_o_^2^) + (0.0589*P*)^2^ + 0.0748*P*] where *P* = (*F*_o_^2^ + 2*F*_c_^2^)/3
2253 reflections	(Δ/σ)_max_ < 0.001
172 parameters	Δρ_max_ = 0.24 e Å^−^^3^
0 restraints	Δρ_min_ = −0.34 e Å^−^^3^

### Special details

**Table:**

Geometry. All esds (except the esd in the dihedral angle between two l.s. planes) are estimated using the full covariance matrix. The cell esds are taken into account individually in the estimation of esds in distances, angles and torsion angles; correlations between esds in cell parameters are only used when they are defined by crystal symmetry. An approximate (isotropic) treatment of cell esds is used for estimating esds involving l.s. planes.
Refinement. Refinement of *F*^2^ against ALL reflections. The weighted *R*-factor wR and goodness of fit *S* are based on *F*^2^, conventional *R*-factors *R* are based on *F*, with *F* set to zero for negative *F*^2^. The threshold expression of *F*^2^ > σ(*F*^2^) is used only for calculating *R*-factors(gt) etc. and is not relevant to the choice of reflections for refinement. *R*-factors based on *F*^2^ are statistically about twice as large as those based on *F*, and *R*- factors based on ALL data will be even larger.

### Fractional atomic coordinates and isotropic or equivalent isotropic displacement parameters (Å^2^)

**Table:**

	*x*	*y*	*z*	*U*_iso_*/*U*_eq_	
Cl2	0.79559 (6)	0.99712 (12)	0.32117 (3)	0.0704 (2)	
Cl1	1.04334 (5)	0.97477 (8)	0.09915 (3)	0.04955 (18)	
O3	0.66872 (11)	0.28902 (19)	−0.04910 (7)	0.0376 (3)	
O2	0.66425 (13)	0.6226 (2)	−0.01578 (8)	0.0476 (4)	
N1	0.49666 (14)	0.1167 (2)	−0.11561 (8)	0.0339 (3)	
N2	0.52884 (17)	0.0126 (3)	−0.17284 (9)	0.0438 (4)	
N3	0.39084 (16)	−0.1726 (3)	−0.12151 (9)	0.0464 (4)	
C1	0.87849 (16)	0.6753 (3)	0.12208 (9)	0.0344 (4)	
C6	0.79694 (17)	0.5867 (3)	0.16172 (10)	0.0415 (5)	
H6A	0.7583	0.4613	0.1466	0.050*	
C4	0.82796 (18)	0.8697 (4)	0.24517 (10)	0.0425 (5)	
C5	0.77190 (18)	0.6821 (4)	0.22373 (10)	0.0451 (5)	
H5A	0.7179	0.6202	0.2506	0.054*	
C3	0.91140 (18)	0.9612 (3)	0.20703 (10)	0.0399 (5)	
H3B	0.9496	1.0869	0.2223	0.048*	
C2	0.93678 (16)	0.8624 (3)	0.14596 (9)	0.0347 (4)	
O1	0.90954 (12)	0.5941 (2)	0.06039 (7)	0.0449 (4)	
C7	0.84682 (18)	0.4108 (3)	0.02987 (11)	0.0427 (5)	
H7A	0.8396	0.3144	0.0687	0.051*	
H7B	0.8964	0.3449	−0.0018	0.051*	
C8	0.71716 (17)	0.4595 (3)	−0.01307 (9)	0.0340 (4)	
C9	0.54632 (17)	0.3176 (3)	−0.09501 (10)	0.0385 (4)	
H9A	0.4909	0.3915	−0.0687	0.046*	
H9B	0.5539	0.3974	−0.1379	0.046*	
C11	0.41456 (18)	0.0036 (3)	−0.08681 (11)	0.0388 (4)	
H11A	0.3792	0.0446	−0.0475	0.047*	
C10	0.4627 (2)	−0.1587 (3)	−0.17314 (11)	0.0470 (5)	
H10A	0.4651	−0.2639	−0.2068	0.056*	

### Atomic displacement parameters (Å^2^)

**Table:**

	*U*^11^	*U*^22^	*U*^33^	*U*^12^	*U*^13^	*U*^23^
Cl2	0.0569 (4)	0.1092 (5)	0.0496 (3)	−0.0030 (3)	0.0214 (3)	−0.0367 (3)
Cl1	0.0582 (3)	0.0474 (3)	0.0477 (3)	−0.0075 (2)	0.0221 (2)	−0.0001 (2)
O3	0.0317 (7)	0.0377 (7)	0.0426 (7)	−0.0016 (6)	0.0049 (5)	−0.0078 (6)
O2	0.0464 (8)	0.0405 (8)	0.0541 (8)	0.0036 (7)	0.0049 (6)	−0.0140 (7)
N1	0.0339 (8)	0.0340 (8)	0.0343 (8)	−0.0040 (7)	0.0081 (6)	−0.0048 (7)
N2	0.0538 (10)	0.0439 (9)	0.0368 (9)	−0.0011 (8)	0.0166 (7)	−0.0047 (7)
N3	0.0482 (10)	0.0400 (9)	0.0501 (10)	−0.0091 (8)	0.0077 (8)	−0.0015 (8)
C1	0.0277 (9)	0.0454 (10)	0.0295 (9)	0.0023 (8)	0.0040 (7)	−0.0079 (8)
C6	0.0319 (10)	0.0476 (11)	0.0447 (11)	−0.0079 (9)	0.0063 (8)	−0.0108 (9)
C4	0.0337 (10)	0.0625 (13)	0.0319 (9)	0.0053 (9)	0.0077 (7)	−0.0116 (9)
C5	0.0304 (10)	0.0656 (13)	0.0406 (10)	−0.0045 (9)	0.0107 (8)	−0.0032 (10)
C3	0.0365 (10)	0.0439 (11)	0.0379 (10)	0.0012 (8)	0.0037 (8)	−0.0105 (8)
C2	0.0309 (9)	0.0391 (10)	0.0336 (9)	0.0012 (8)	0.0053 (7)	−0.0015 (8)
O1	0.0374 (7)	0.0564 (8)	0.0436 (7)	−0.0089 (7)	0.0145 (6)	−0.0223 (7)
C7	0.0360 (10)	0.0464 (11)	0.0457 (11)	−0.0004 (9)	0.0075 (8)	−0.0202 (9)
C8	0.0350 (10)	0.0394 (10)	0.0312 (9)	−0.0045 (8)	0.0155 (7)	−0.0090 (8)
C9	0.0328 (10)	0.0351 (9)	0.0468 (11)	−0.0031 (8)	0.0058 (8)	−0.0030 (8)
C11	0.0361 (10)	0.0435 (10)	0.0380 (10)	−0.0053 (8)	0.0108 (8)	−0.0006 (8)
C10	0.0603 (13)	0.0379 (10)	0.0420 (11)	−0.0002 (10)	0.0074 (9)	−0.0081 (9)

### Geometric parameters (Å, °)

**Table:**

Cl2—C4	1.7346 (19)	C6—H6A	0.9300
Cl1—C2	1.7336 (19)	C4—C5	1.378 (3)
O3—C8	1.343 (2)	C4—C3	1.385 (3)
O3—C9	1.445 (2)	C5—H5A	0.9300
O2—C8	1.194 (2)	C3—C2	1.380 (3)
N1—C11	1.339 (2)	C3—H3B	0.9300
N1—N2	1.364 (2)	O1—C7	1.427 (2)
N1—C9	1.427 (2)	C7—C8	1.509 (3)
N2—C10	1.316 (3)	C7—H7A	0.9700
N3—C11	1.310 (3)	C7—H7B	0.9700
N3—C10	1.353 (3)	C9—H9A	0.9700
C1—O1	1.366 (2)	C9—H9B	0.9700
C1—C6	1.380 (3)	C11—H11A	0.9300
C1—C2	1.395 (3)	C10—H10A	0.9300
C6—C5	1.384 (3)		
			
C8—O3—C9	114.73 (14)	C1—C2—Cl1	120.23 (14)
C11—N1—N2	109.61 (15)	C1—O1—C7	118.73 (15)
C11—N1—C9	129.08 (16)	O1—C7—C8	111.21 (16)
N2—N1—C9	121.29 (16)	O1—C7—H7A	109.4
C10—N2—N1	101.33 (16)	C8—C7—H7A	109.4
C11—N3—C10	102.19 (16)	O1—C7—H7B	109.4
O1—C1—C6	125.49 (17)	C8—C7—H7B	109.4
O1—C1—C2	115.65 (16)	H7A—C7—H7B	108.0
C6—C1—C2	118.86 (17)	O2—C8—O3	124.25 (16)
C1—C6—C5	120.90 (19)	O2—C8—C7	126.51 (17)
C1—C6—H6A	119.5	O3—C8—C7	109.23 (15)
C5—C6—H6A	119.5	N1—C9—O3	107.39 (15)
C5—C4—C3	121.22 (17)	N1—C9—H9A	110.2
C5—C4—Cl2	120.53 (16)	O3—C9—H9A	110.2
C3—C4—Cl2	118.25 (16)	N1—C9—H9B	110.2
C4—C5—C6	119.20 (19)	O3—C9—H9B	110.2
C4—C5—H5A	120.4	H9A—C9—H9B	108.5
C6—C5—H5A	120.4	N3—C11—N1	110.84 (18)
C2—C3—C4	118.78 (18)	N3—C11—H11A	124.6
C2—C3—H3B	120.6	N1—C11—H11A	124.6
C4—C3—H3B	120.6	N2—C10—N3	116.03 (17)
C3—C2—C1	121.00 (18)	N2—C10—H10A	122.0
C3—C2—Cl1	118.77 (15)	N3—C10—H10A	122.0
			
C11—N1—N2—C10	−0.5 (2)	C6—C1—O1—C7	5.1 (3)
C9—N1—N2—C10	−179.25 (16)	C2—C1—O1—C7	−175.59 (16)
O1—C1—C6—C5	179.94 (17)	C1—O1—C7—C8	78.8 (2)
C2—C1—C6—C5	0.7 (3)	C9—O3—C8—O2	2.2 (3)
C3—C4—C5—C6	−1.9 (3)	C9—O3—C8—C7	−177.47 (15)
Cl2—C4—C5—C6	178.27 (16)	O1—C7—C8—O2	−8.3 (3)
C1—C6—C5—C4	1.1 (3)	O1—C7—C8—O3	171.40 (15)
C5—C4—C3—C2	0.9 (3)	C11—N1—C9—O3	98.2 (2)
Cl2—C4—C3—C2	−179.26 (14)	N2—N1—C9—O3	−83.4 (2)
C4—C3—C2—C1	0.9 (3)	C8—O3—C9—N1	−167.03 (15)
C4—C3—C2—Cl1	−178.88 (14)	C10—N3—C11—N1	−0.4 (2)
O1—C1—C2—C3	178.97 (17)	N2—N1—C11—N3	0.6 (2)
C6—C1—C2—C3	−1.7 (3)	C9—N1—C11—N3	179.20 (17)
O1—C1—C2—Cl1	−1.2 (2)	N1—N2—C10—N3	0.3 (2)
C6—C1—C2—Cl1	178.12 (15)	C11—N3—C10—N2	0.0 (2)

### Hydrogen-bond geometry (Å, °)

**Table:**

*D*—H···*A*	*D*—H	H···*A*	*D*···*A*	*D*—H···*A*
C6—H6A···N3^i^	0.93	2.45	3.353 (3)	164
C7—H7B···O1^ii^	0.97	2.58	3.390 (2)	142
C9—H9A···O2^iii^	0.97	2.52	3.381 (3)	148
C11—H11A···O2^iii^	0.93	2.54	3.293 (2)	139
Cg1—···.Cg2^i^	.	.	3.665 (2)	.

Symmetry codes: (i) −*x*+1, −*y*, −*z*; (ii) −*x*+2, −*y*+1, −*z*; (iii) −*x*+1, −*y*+1, −*z*.
